# Endocrine and systemic toxicity of pyriproxyfen in adult female zebrafish: disruption of the HPT axis and peripheral organ impairments

**DOI:** 10.1007/s11356-026-38319-1

**Published:** 2026-10-19

**Authors:** Andressa Pereira Cabral, Fabricio Pereira dos Santos Maia, Damáris Barcelos Cunha Azeredo, Andréa Souza de Jesus Santana, Lycia de Brito Gitirana, John Lennon de Paiva Coimbra, Denise Pires de Carvalho, Paula Soares, Eduardo Rios-Morris, Leandro Miranda-Alves

**Affiliations:** 1 Institute of Biomedical Sciences, Federal University of Rio de Janeiro, Rio de Janeiro, Brazil; 2 Institute of Biophysics Carlos Chagas Filho, Federal University of Rio de Janeiro, Rio de Janeiro, Brazil; 3 Cancer Signaling and Metabolism Group, i3S – Institute for Research and Innovation in Health, University of Porto, Porto, Portugal; 4 Department of Pathology, Faculty of Medicine, University of Porto, Porto, Portugal; 5 Laboratory of Experimental Endocrinology-LEEx, Federal University of Rio de Janeiro, Rio de Janeiro, Brazil

**Keywords:** Endocrine disruptor, Juvenile hormone, Pesticide, Pyriproxyfen, Zebrafish, Ecotoxicology

## Abstract

Endocrine-disrupting chemicals (EDCs) can destabilize the hypothalamic–pituitary–thyroid (HPT) axis, a fundamental regulator of vertebrate development and metabolism. Pyriproxyfen (PPF), a juvenile hormone analog widely utilized in vector control, is recommended by the World Health Organization (WHO) for application at concentrations of up to 10 µg/L in drinking water; however, its safety profile for vertebrates remains a matter of concern. In this study, we investigated the endocrine and systemic impacts of PPF in adult female zebrafish exposed for 14 days to 1, 10, or 100 µg/L. Histological, hormonal, and transcriptional analyses across the thyroid, brain, liver, and kidney revealed a non-monotonic response centered on the WHO reference application concentration. The 10 µg/L group exhibited the most significant molecular impairment in the thyroid, characterized by the upregulation of *slc5a5* (*nis*), *nkx2.1*, *pax2a*, and *tshr*, while repressing thyroglobulin (*tg)* expression. Consistent with these molecular alterations, thyroid epithelial disruption was observed at 1 and 10 µg/L. In contrast, exposure to 100 µg/L increased plasma TSH levels and the expression of pituitary *tshb* and hepatic *dio1,* suggesting a compensatory response of the HPT axis. Renal morphometry additionally revealed tubular alterations across all exposure groups. Overall, these findings demonstrate a non-monotonic concentration–response centered on 10 µg/L as a key mechanistic feature of PPF toxicity and support further evaluation of thyroid and renal endpoints in ecotoxicological risk assessment.

**Supplementary Information:**

The online version contains supplementary material available at https://doi.org/10.1007/s11356-026-38319-1.

## Introduction

In an era defined by anthropogenic pressures on delicate ecosystems, the pervasive presence of environmental contaminants demands rigorous scientific scrutiny (Pereira et al. 2015; Raza et al. 2024). Many of these pollutants are persistent, bioaccumulative, and capable of causing a wide range of health problems depending on the duration and level of exposure (Megson et al. 2016; He et al. 2024). Among these, endocrine-disrupting chemicals (EDCs) represent a particularly insidious threat, capable of subtly yet profoundly altering physiological processes essential for life (Kamarudin et al. 2020; Ahn and Jeung 2023; Río Barrera et al. 2025).

The hypothalamic-pituitary-thyroid (HPT) axis, a major regulator of development, metabolism, and homeostasis across vertebrates, represents a critical target for such compounds (Ikegami and Yoshimura 2017; Paul et al. 2022). Growing evidence unequivocally links EDC exposure to both direct dysfunction of the HPT axis and a spectrum of adverse health outcomes including metabolic disorders (Schmutzler et al. 2007; Yilmaz et al. 2020), reproductive disorders, obesity, and neurodevelopmental alterations, underscoring their profound relevance to both environmental integrity and human well-being (Jugan et al. 2010; Egalini et al. 2022; Paiva-Melo et al. 2025; Azeredo et al. 2025).

Pest control, both in agriculture for crop protection and in public health, relies extensively on the use of pesticides (Tudi et al. 2021). These compounds represent an important tool in combating vectors of arboviruses, such as the mosquito *Aedes aegypti* (Ur Rahman et al. 2024), responsible for the transmission of major diseases including dengue, Zika, and chikungunya (Monteiro et al. 2020). In this context, pyriproxyfen (PPF) stands out as one of the most widely employed compounds (Hustedt et al. 2020).

PPF acts as an analog of juvenile hormone (JH), an essential endogenous regulator in insects that controls metamorphosis (Alomar and Alto 2022). The presence of this JH analog prevents larvae from progressing to the adult stage (Hustedt et al. 2020). By mimicking this function, PPF disrupts the vector’s life cycle before it reaches maturity and becomes capable of transmitting diseases (Yadav et al. 2019; Grisales et al. 2021).

Despite its effectiveness, the extensive use of PPF has led to its increasing detection in the environment. As a result, residues of the compound have been detected in multiple environmental compartments (Xu et al. 2024), including soil and rivers (Devillers 2020a; Kumari et al. 2021). Residues have also been reported in food-related matrices such as honey and other bee products (Wu et al. 2021) and PPF has been shown to bioconcentrate in non-target aquatic organisms (Moura and Souza-Santos 2020; Devillers 2020b), including crustaceans and fish (Abdu et al. 2001; Ginjupalli and Baldwin 2013; Hustedt et al. 2021; Robea et al. 2023). In zebrafish, reported bioconcentration factors (BCFs) of 1175–1246 remain below the regulatory threshold used to classify a substance as bioaccumulative (BCF > 2000) (Wei et al. 2021). More directly, and of greater concern, in some regions PPF is applied directly to human drinking water reservoirs, such as household water tanks, for *Aedes aegypti* larval control (World Health Organization 2008; Truong et al. 2016). This combination of diffuse environmental contamination and direct exposure increases the likelihood of exposure of ecosystems, and human populations to PPF, underscoring the urgency of reassessing the toxicological safety of this compound.

The zebrafish (*Danio rerio*) has established itself as a reference experimental model in ecotoxicology and endocrinology due to its unique combination of biological and experimental advantages (Segner 2009; Teame et al. 2019; Porto et al. 2023). It exhibits strong genomic conservation with humans; approximately 70% of genes have human orthologs, including more than 80% of disease-related genes, supporting its utility for investigating conserved biological mechanisms (Adhish and Manjubala 2023). This conservation extends critically to regulatory axes such as the HPT axis, making zebrafish particularly valuable for investigations of endocrine disruption (Segner 2009; Baumann et al. 2016). Furthermore, its sensitivity to environmental contaminants and applicability in high-throughput assays reinforce zebrafish as an important model for evaluating the toxicological impacts of chemical substances.

Despite the recognized importance of understanding EDCs’ impacts on the HPT axis, a comprehensive and integrated perspective on PPF’s effects across multiple tissues remains elusive. Recent evidence shows that exposure to PPF (Horie et al. 2023) and its primary metabolite, 4′-OH-PPF (Vancamp et al. 2021), can interfere with thyroid hormone signaling and related developmental processes. Together, these studies indicate that PPF and its primary metabolite can disrupt the regulation of genes involved in growth and neurodevelopment, including those associated with thyroid hormone biosynthesis and signaling, suggesting that PPF exposure, through both the parent compound and its metabolite, may act as a thyroid hormone disruptor affecting multiple biological systems (Vancamp et al. 2021; Horie et al. 2023). Complementing these observations, Maharajan et al. (2020) reported that exposure of adult zebrafish to pyriproxyfen impairs reproductive endocrine homeostasis and gonadal architecture through altered expression of hypothalamic-pituitary–gonadal axis genes. To bridge these critical knowledge gaps, the present study meticulously investigates the effects of varying PPF concentrations on HPT axis gene expression in zebrafish across three pivotal tissues: the thyroid, brain, and liver.

Thyroid hormone homeostasis is finely regulated at the tissue level by an important family of enzymes, the deiodinases (DIOs) (Köhrle 2000; Sabatino et al. 2021). These enzymes mediate hormone activation and inactivation, playing a crucial role in regulating local hormone availability (Luongo et al. 2019). DIO1 and DIO2 are responsible for converting the relatively inactive prohormone, thyroxine (T4), into its biologically active form, triiodothyronine (T3) (Luongo et al. 2019; Köhrle and Frädrich 2022). Conversely, DIO3 catalyzes the inactivation of both T4 and T3, thereby terminating their biological activity (Gereben et al. 2008). Therefore, the analysis of these enzymes is fundamental to understanding the peripheral regulation of thyroid signaling.

This comprehensive approach aims to deepen our understanding of how PPF exposure may disrupt thyroid hormone homeostasis. We assessed peripheral thyroid function markers (*nis*, *tshr*, *tg*, *tpo*, *pax2a*, and *nkx2.1*) (Ely et al. 2018; Cazarin et al. 2022), central regulatory genes (*trh*, *trhr*, *thra*, and *tshb*) (Chiamolera et al. 2012), and liver-associated mediators of thyroid hormone metabolism and stress responses (*dio1*, *dio2*, *nrf2*, *sod3*, and *tnfα*) (Jiao et al. 2019; Russo et al. 2021). By examining PPF’s impact on central regulatory pathways, peripheral hormone synthesis and conversion, and antioxidant defense mechanisms, this study provides meaningful insights into the ecological toxicology. Ultimately, it seeks to clarify the broader environmental and health implications of PPF exposure.

## Materials and methods

### Chemicals

Pyriproxyfen (PPF; PESTANAL®, analytical standard, purity ≥ 98%; CAS no. 95737–68-1; cat. no. 34174; Sigma-Aldrich, St. Louis, MO, USA) was used as the test compound. A stock solution was prepared by dissolving PPF in analytical-grade dimethyl sulfoxide (DMSO, Neon, Brazil) at 1% (v/v). The stock solution was prepared before the beginning of the experiment and stored under refrigeration in an amber glass bottle, protected from light throughout the 14-day exposure period. Fresh exposure solutions were prepared daily by diluting the stock solution in system water at each renewal. The final concentration of DMSO in the exposure tanks did not exceed 0.01% (v/v), a level considered non-toxic for zebrafish and included in all control treatments (Christou et al. 2020; Hoyberghs et al. 2021).

### Animals and housing

The study was conducted at the Unit of (Eco)Toxicology of the Laboratory of Experimental Endocrinology, Institute of Biomedical Sciences, Federal University of Rio de Janeiro (UFRJ), Brazil. The facility is certified by the Coordination of Activities with Experimental Biological Models (CAMBE).

Wild-type zebrafish (*Danio rerio*) were obtained from a commercial supplier (Carlao Peixes Ornamentais, Rio de Janeiro, Brazil) and maintained at the (Eco)Toxicology Unit until the beginning of the experiments, at approximately 3–4 months of age. Only adult females were selected for the experiments. Individual body length and body mass were not recorded for the experimental cohort.

Prior to experimental acclimation, fish were maintained in a Hydrus recirculating aquaculture system (Alesco, Brazil), in 3-L tanks containing up to 15 adult fish (approximately 5 fish/L). System water underwent mechanical, chemical, and biological filtration, and daily ultraviolet sterilization. During the rearing and acclimation, water parameters were monitored daily and maintained at 28 ± 1 °C, a conductivity of approximately 600 µS/cm, and pH 7.2, under a 14 h light/10 h dark photoperiod. Animals were fed to apparent satiety three times daily: twice with commercial fish feed (Alcon Basic®, Brazil) and once with freshly hatched *Artemia nauplii* produced in the laboratory. The experimental animals were acclimated for 7 days under these same conditions before exposure.

## Experimental design and exposure

**Fig. 1:**
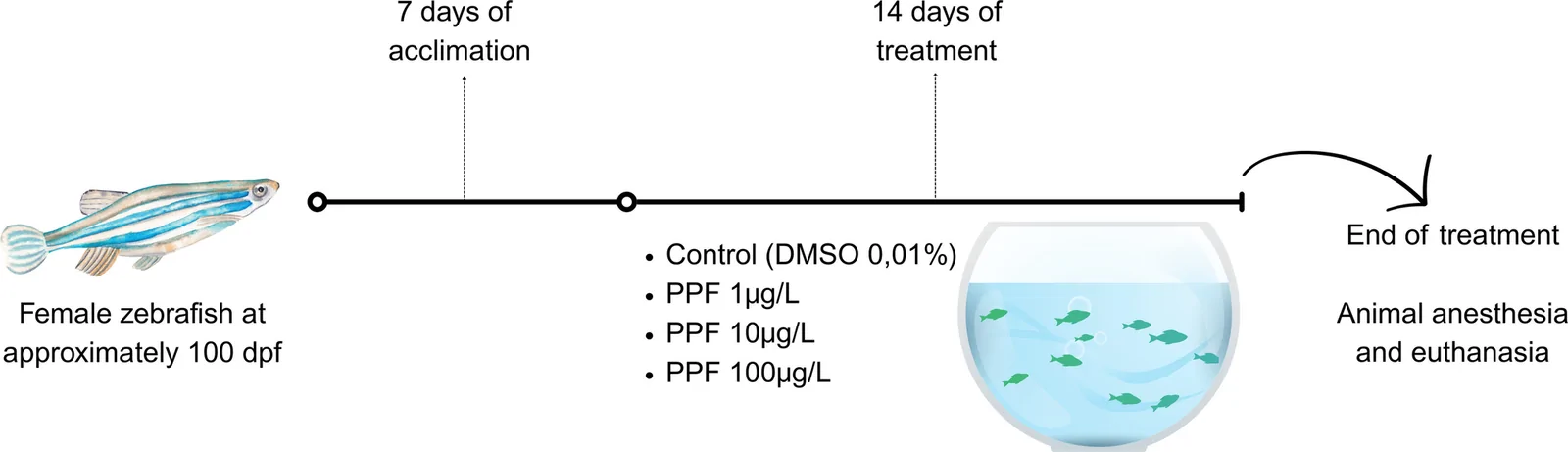
Representative scheme of the experimental design used in this work. Female zebrafish were exposed to PPF at three different concentrations (1, 10, and 100 μg/L). This fig. was made using the Canva platform

The experimental protocol consisted of four groups: control, 1 µg/L PPF, 10 µg/L PPF, and 100 µg/L PPF (Fig. 1). The test concentrations were selected as a logarithmic series centered at 10 µg/L, the maximum application concentration recommended by the WHO for pyriproxyfen application in potable-water containers (World Health Organization 2008). The concentrations of 1 and 100 µg/L represent one order of magnitude below and above this reference concentration, respectively. The exposure medium consisted of the same treated system of water used for rearing and acclimation, supplemented with either the appropriate PPF working solution or the vehicle. Control groups received the same final concentration of DMSO (≤ 0.01%, v/v) used as a vehicle in the treated groups.

Exposures were conducted for 14 consecutive days under semi-static conditions, with complete daily renewal of the exposure solutions. Ten females were maintained in each glass aquarium containing 2 L of exposure medium, with continuous aeration in a biochemical oxygen demand (BOD) incubator. Temperature, pH, conductivity, and dissolved oxygen were monitored throughout the exposure period and checked at each daily renewal.

At the end of the exposure period, animals were deeply anesthetized using tricaine methanesulfonate (MS-222, Sigma-Aldrich) and euthanized by rapid immersion in ice-cold water, in accordance with the American Veterinary Medical Association Guidelines for the Euthanasia of Animals (2020), the European Parliament and Council (2010) Directive 2010/63/EU, and the National Council for the Control of Animal Experimentation – CONCEA (2017) recommendations.

Following euthanasia, blood samples were collected by severing the caudal peduncle. Blood was collected into 1.5 mL microtubes and centrifuged at 2000 rpm for 90 s to obtain plasma, which was stored at − 80 °C until hormone analysis. Whole tissues (thyroid, brain, liver, and kidney) were dissected immediately after blood collection, processed individually or pooled depending on the subsequent analysis.

Sample sizes varied according to the type of assay:

- Histology: three independent replicates with three animals per group.
- Molecular analyses (qPCR): three biological replicates, each consisting of pooled tissue from 10 animals per group.
- Hormonal assays: three independent experimental replicates were performed for each treatment group. In each replicate, pooled blood samples were obtained from 50 animals.

### Sample collection and processing

#### Histology and morphometric analysis

Animals used for histological analysis were prepared to improve fixative penetration. The caudal peduncle was severed, and small incisions were made in the abdominal region. Whole animals were fixed in Bouin’s solution for approximately 3 h at room temperature, rinsed with 70% ethanol, and subsequently decalcified in 8% ethylenediaminetetraacetic acid (EDTA, pH 7.4) for 7 days, with three solution changes. Samples were then processed in an automated tissue processor (Leica TP1020, Leica Microsystems, Germany), dehydrated through a graded ethanol series, cleared in xylene, and embedded in paraffin. Serial sections of 5 µm thickness were obtained using a rotary microtome and mounted on glass slides.

For histological evaluation, sections were stained with hematoxylin and eosin (H&E) and the periodic acid–Schiff (PAS) staining. Analyses and acquisition of representative images (2048 × 1536 pixels) were performed on an Olympus B201 optical microscope equipped with a Retiga 2000R digital camera (QImaging, Vancouver, BC, Canada).

Morphometric evaluation of thyroid follicles was conducted using ImageJ software (NIH, Bethesda, MD, USA). For each group, at least 30 follicles were analyzed, and the following parameters were measured: follicle height and width, colloid height and width, follicular area, and colloid area. Epithelial height was estimated by subtracting colloid from follicle dimensions. Additionally, the epithelial area was calculated as the difference between follicle and colloid areas, and the epithelium-to-colloid ratio was calculated as the follicular epithelial area divided by the colloid area. Measurements were performed in three biological replicates per group, and results were expressed as mean values per animal (Wade et al. 2002; Bianco et al. 2014).

### Molecular analyses (qPCR)

Total RNA was extracted from thyroid-enriched tissue, brain, and liver tissues using the RNeasy® Plus Mini Kit (QIAGEN, TX, USA). RNA concentration and purity were assessed with a NanoDrop spectrophotometer (Thermo Fisher Scientific, CA, USA). Complementary DNA (cDNA) was synthesized from 1 μg RNA using the High-Capacity cDNA Reverse Transcription Kit (Applied Biosystems, CA, USA). qPCR was performed on a 7500 Real-Time PCR System (Applied Biosystems, CA, USA) with GoTaq® qPCR Master Mix (Promega, WI, USA). Each sample was amplified in technical duplicates, and β-actin (actb1) was used as the endogenous control. Primers were designed and validated in-house, based on published zebrafish sequences, and are listed in Table S1 (Supplementary material). Relative expression of target genes was calculated using the 2^⁻ΔΔCt^ method (Livak and Schmittgen 2001). Analyses were performed with three independent biological replicates, each consisting of pooled tissues from 10 animals per group.

### Hormonal quantification

Plasma concentrations of thyroxine (T4) and thyroid-stimulating hormone (TSH) were quantified using commercial enzyme-linked immunosorbent assay (ELISA) kits (Cusabio Biotech Co; T4: CSB-E08489f; TSH: CSB-EQ027261FI). Plasma samples were diluted 1:10 in phosphate-buffered saline (PBS, pH 7.4), as preliminary tests confirmed that diluted values fell within the standard curves provided by the manufacturer (Lee et al. 2020; Ihn et al. 2024).

All procedures were carried out according to the manufacturer’s instructions. Optical density was measured using a SpectraMax® multimode fluorescence microplate reader (Molecular Devices, San Jose, CA, USA), and hormone concentrations were calculated based on standard curves generated for each assay. Results were expressed as mean concentrations from three independent biological replicates, each consisting of pooled plasma from 50 animals per treatment group.

### Statistical analysis

Results were expressed as the mean ± standard error of the mean (SEM). Statistical analyses were performed using GraphPad Prism 9 (GraphPad Software, Inc., San Diego, California, USA). Data normality and homoscedasticity were assessed using Shapiro–Wilk and Brown-Forsythe tests, respectively. For parametric data, including histomorphometric parameters and relative mRNA expression levels, statistical significance was determined using one-way ANOVA followed by Dunnett's multiple comparisons test to compare treated groups against the control. For data with a non-Gaussian distribution (plasma hormone concentrations), the Kruskal–Wallis test followed by Dunn’s multiple comparisons test was applied. Statistical significance was defined as *p* < 0.05, with higher levels of significance indicated as **p < 0.01, ***p < 0.001, or ****p < 0.0001.

## Results

**Fig. 2:**
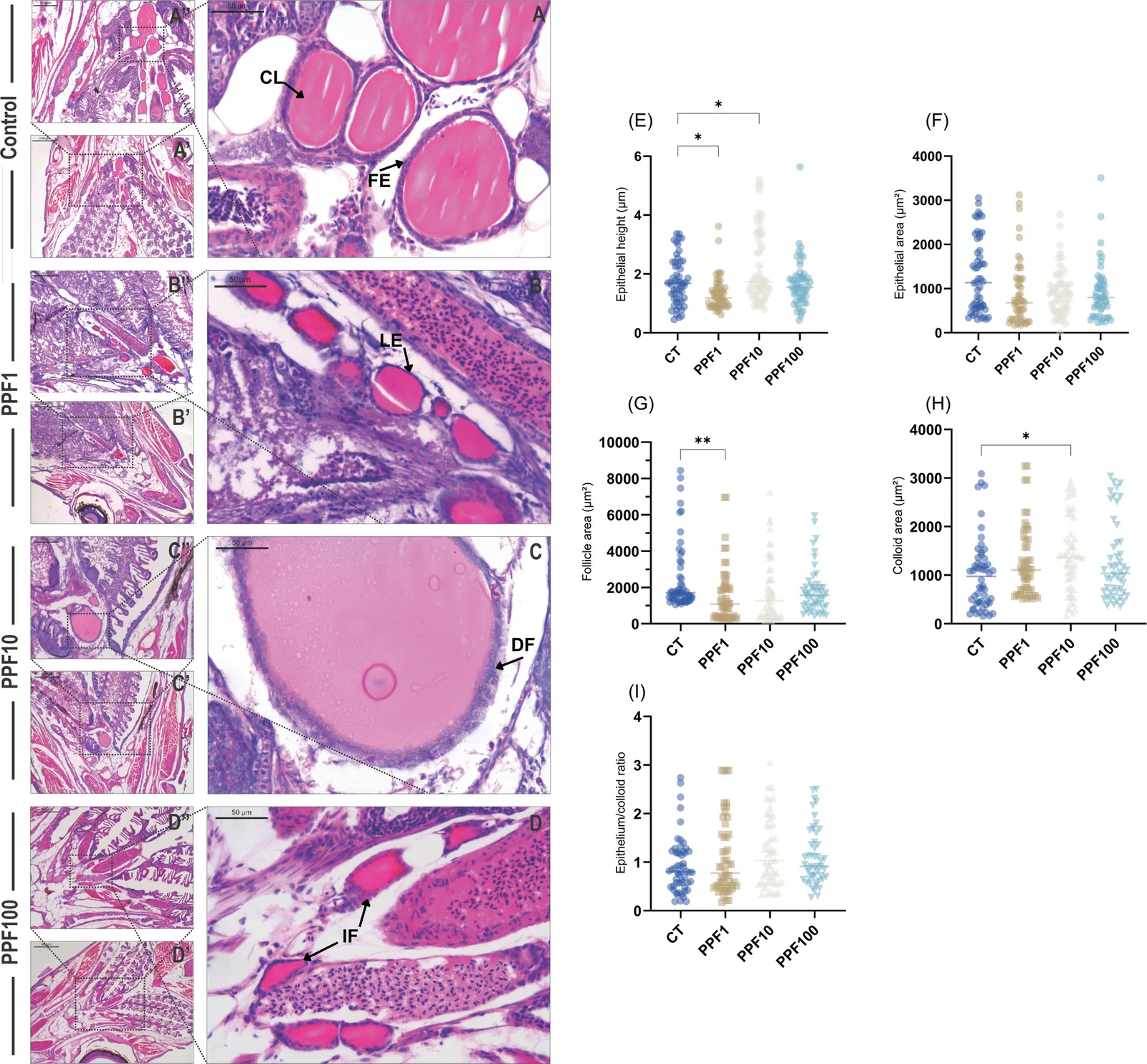
Histological and morphometric analysis of thyroid follicles in adult female zebrafish after 14 days of PPF exposure. Representative H&E stained sections from the control (A-A″), PPF1 (B-B″), PPF10 (C–C″), and PPF100 (D-D″) groups. Arrows in the high-magnification panels identify CL, colloid; FE, follicular epithelium; LE, low/flattened follicular epithelium; DF, dilated follicle; and IF, irregular follicular profiles. Scale bars: 500 μm (A″, B″, C″, and D″), 200 μm (A′, B′, C′, and D′), and 50 μm (**A**, **B**, **C**, and **D**). Morphometric analyses are shown for epithelial height (**E**), epithelial area (**F**), follicle area (**G**), colloid area (**H**), and epithelium-to-colloid area ratio (**I**). Each point represents one follicle. At least 30 follicles were evaluated per group. Data are presented as mean ± SEM. *p < 0.05 and **p < 0.01 compared with the control group (one-way ANOVA followed by Dunnett’s multiple-comparisons test)

Histological and morphometric analyses of thyroid follicles after 14 days of PPF exposure revealed concentration-specific alterations (Fig. 2). In control animals, thyroid follicles were well organized, with homogeneous colloid (CL) surrounded by a simple cuboidal follicular epithelium (FE; Fig. 2a). In the PPF1 group, representative sections showed low/flattened follicular epithelium (LE; Fig. 2b). Morphometric analysis showed significant reductions in epithelial height in the PPF1 and PPF10 groups (Fig. 2e; *p* < 0.05) and in follicle area in the PPF1 group (Fig. 2g; *p* < 0.01) compared with the control group. In the PPF10 group, the representative section showed a dilated follicle (DF; Fig. 2c), accompanied by a significant increase in colloid area (Fig. 2h; *p* < 0.05). In the PPF100 group, representative sections showed irregular follicular profiles (IF; Fig. 2d), indicating qualitative heterogeneity in follicular architecture; however, no significant differences were detected in the measured morphometric parameters compared with the control group (Fig. 2e-i).

### Thyroid hormone concentrations in plasma

**Fig. 3:**
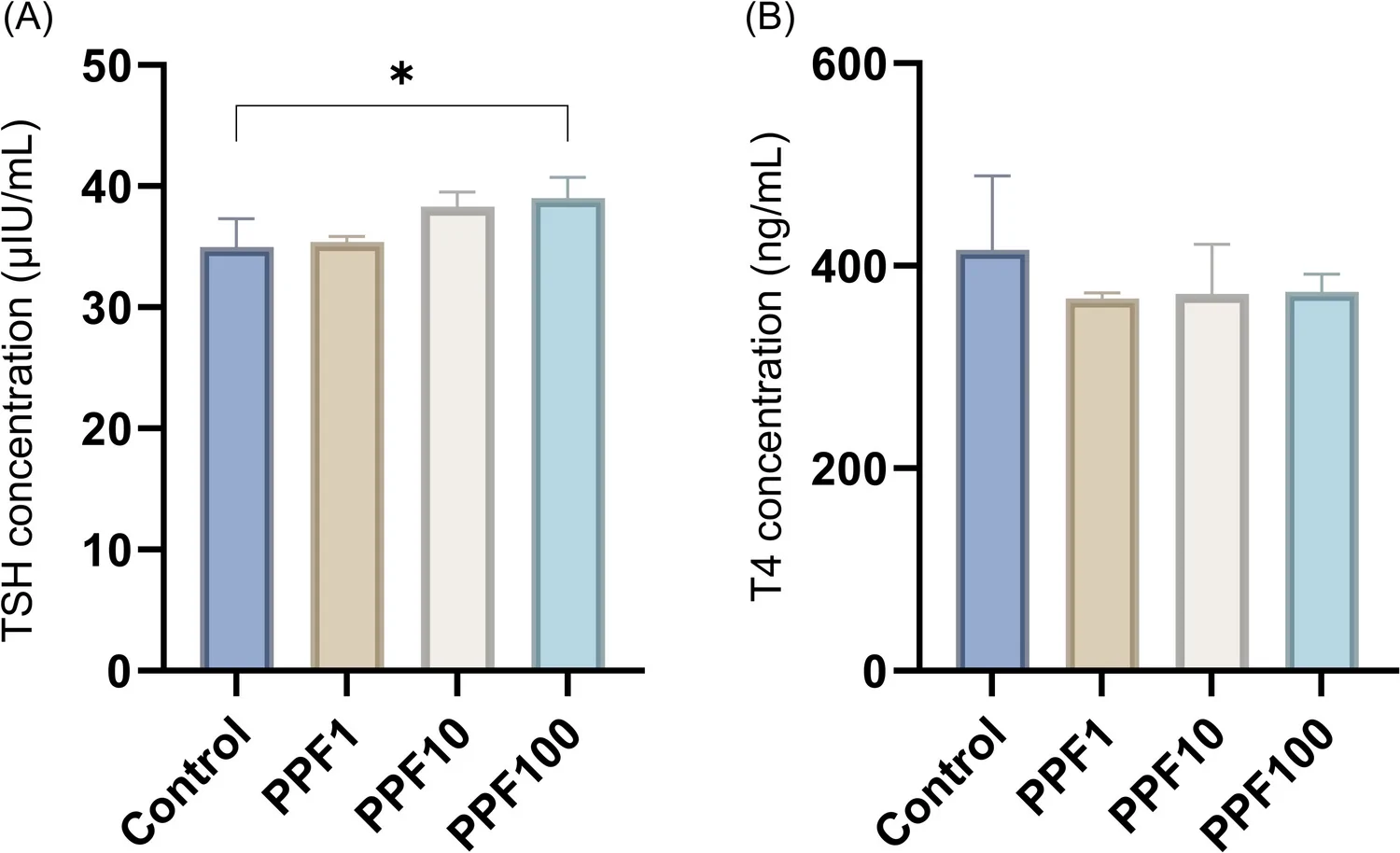
Thyroid hormone concentrations in plasma of adult female zebrafish after 14 days of PPF exposure. Plasma levels of TSH (**a**) and T4 (**b**) were measured following exposure to 1, 10, and 100 µg/L PPF. A significant increase in TSH concentration was observed in fish exposed to 100 µg/L PPF compared with the control group, while T4 levels remained unchanged across all treatment groups. Data are presented as mean ± SEM of three independent biological replicates, with each replicate consisting of pooled plasma from 50 animals. Statistical significance was determined using the Kruskal–Wallis test followed by Dunn’s multiple comparisons test. *p < 0.05 versus control

Plasma concentrations of TSH and T4 were quantified following 14 days of PPF exposure in adult female zebrafish (Fig. 3). TSH concentration significantly increased in groups exposed to 100 μg/L PPF compared to the control group. However, PPF exposure did not induce significant changes in T4 levels at any of the concentrations tested.

### The expression of thyroid genes was analyzed by qPCR

**Fig. 4:**
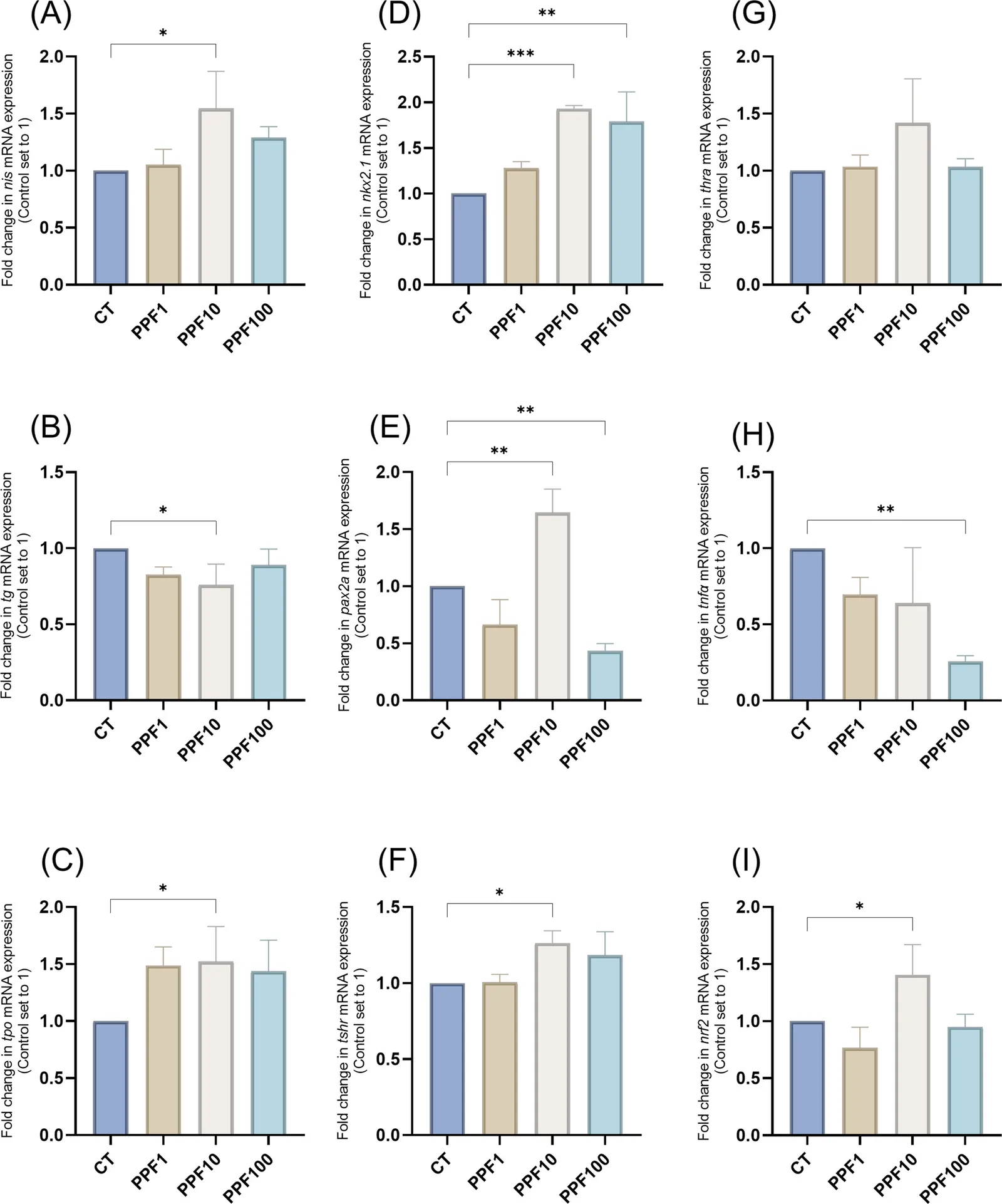
Relative mRNA expression of thyroid-related genes in zebrafish thyroid tissue after 14 days of exposure to PPF. The analyzed genes included (**a**) *slc5a5 (nis)*, sodium/iodide symporter; (**b**) *tg*, thyroglobulin; (**c**) *tpo*, thyroid peroxidase; (**d**) *nkx2.1*, NK2 homeobox 1; (**e**) *pax2a*, paired box 2a; (**f**) *tshr*, thyroid-stimulating hormone receptor; (**g**) *thra*, thyroid hormone receptor alpha; (**h**) *tnfα*, tumor necrosis factor alpha; and (**i**) *nrf2*, nuclear factor erythroid 2-related factor 2. Data are presented as fold change relative to the control group and normalized to actb1. Results are expressed as mean ± SEM of three independent biological replicates (n = 10 fish per replicate). Statistical significance was determined using one-way ANOVA followed by Dunnett's multiple comparisons test. Asterisks indicate significant differences compared with the control group (*p < 0.05; **p < 0.01; ***p < 0.001)

Given the histological alterations observed, we next evaluated whether PPF affected thyroid molecular markers. For this purpose, the expression of key thyroid-related genes was quantified by qPCR (Fig. 4). Compared with the control group, zebrafish exposed to PPF10 exhibited a significant downregulation of *tg* (Fig. 4b), whereas *slc5a5* (*nis*, Fig. 4a), *tpo* (Fig. 4c), *nkx2.1* (Fig. 4d), *pax2a* (Fig. 4e), and *tshr* (Fig. 4f) were significantly upregulated, indicating altered iodide uptake, and thyroid cell specification and differentiation. In addition, PPF exposure did not significantly affect *thra* expression (Fig. 4g). However, *tnfα* was downregulated at the highest PPF concentration (Fig. 4h), while *nrf2* expression increased at the intermediate concentration (Fig. 4i). These findings suggest a potential crosstalk among thyroid function, inflammatory response, and oxidative stress pathways. Collectively, these results indicate that PPF disrupts thyroid functional capacity at the molecular level.

### The expression of central regulators of the hypothalamic-pituitary-thyroid (HPT) axis was evaluated in the brain

**Fig. 5:**
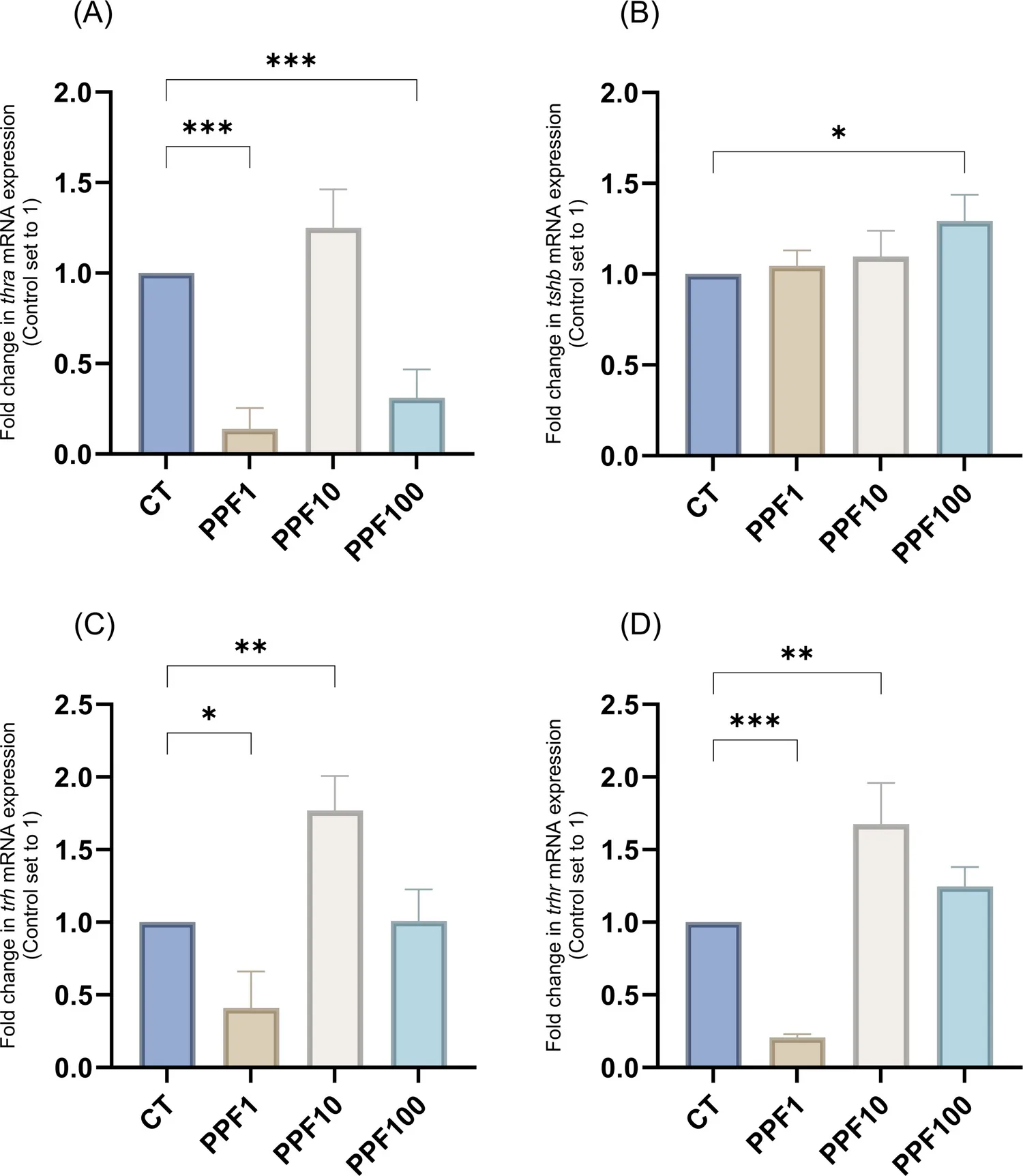
Relative mRNA expression of central regulators of the HPT axis in zebrafish brain, determined by qPCR. Data are presented as fold change relative to the control group, normalized to *actb1*. (**a**) *thra*, thyroid hormone receptor alpha; (**b**) *tshb*, thyroid-stimulating hormone beta subunit; (**c**) *trh*, thyrotropin-releasing hormone; and (**d**) *trhr*, thyrotropin-releasing hormone receptor. Values represent mean ± SEM of three independent biological replicates (n = 10 fish per replicate). Statistical significance was determined using one-way ANOVA followed by Dunnett's multiple comparisons test. Asterisks indicate significant differences compared with the control group (*p < 0.05; **p < 0.01; ***p < 0.001)

The expression of key regulatory genes of the HPT axis in the zebrafish brain revealed a complex, non-monotonic regulatory response across PPF concentrations (Fig. 5). At the lowest concentration (PPF1), a significant downregulation was observed for *thra* (Fig. 5a), *trh* (Fig. 5c), and *trhr* (Fig. 5d). In contrast, the PPF10 group exhibited a non-monotonic response, characterized by a significant upregulation of *trh* (Fig. 5c) and *trhr* (Fig. 5d) compared with the control, and the opposing responses at 1 and 10 µg/L were consistent with a non-monotonic pattern. At the highest concentration (PPF100), while *thra* mRNA expression remained significantly suppressed (Fig. 5a), there was a significant increase in *tshb* expression (Fig. 5b), suggesting a compensatory stimulation of the thyroid axis at the pituitary level under higher PPF concentrations.

### The expression of thyroid hormone metabolism and stress-related genes was assessed in the liver

**Fig. 6:**
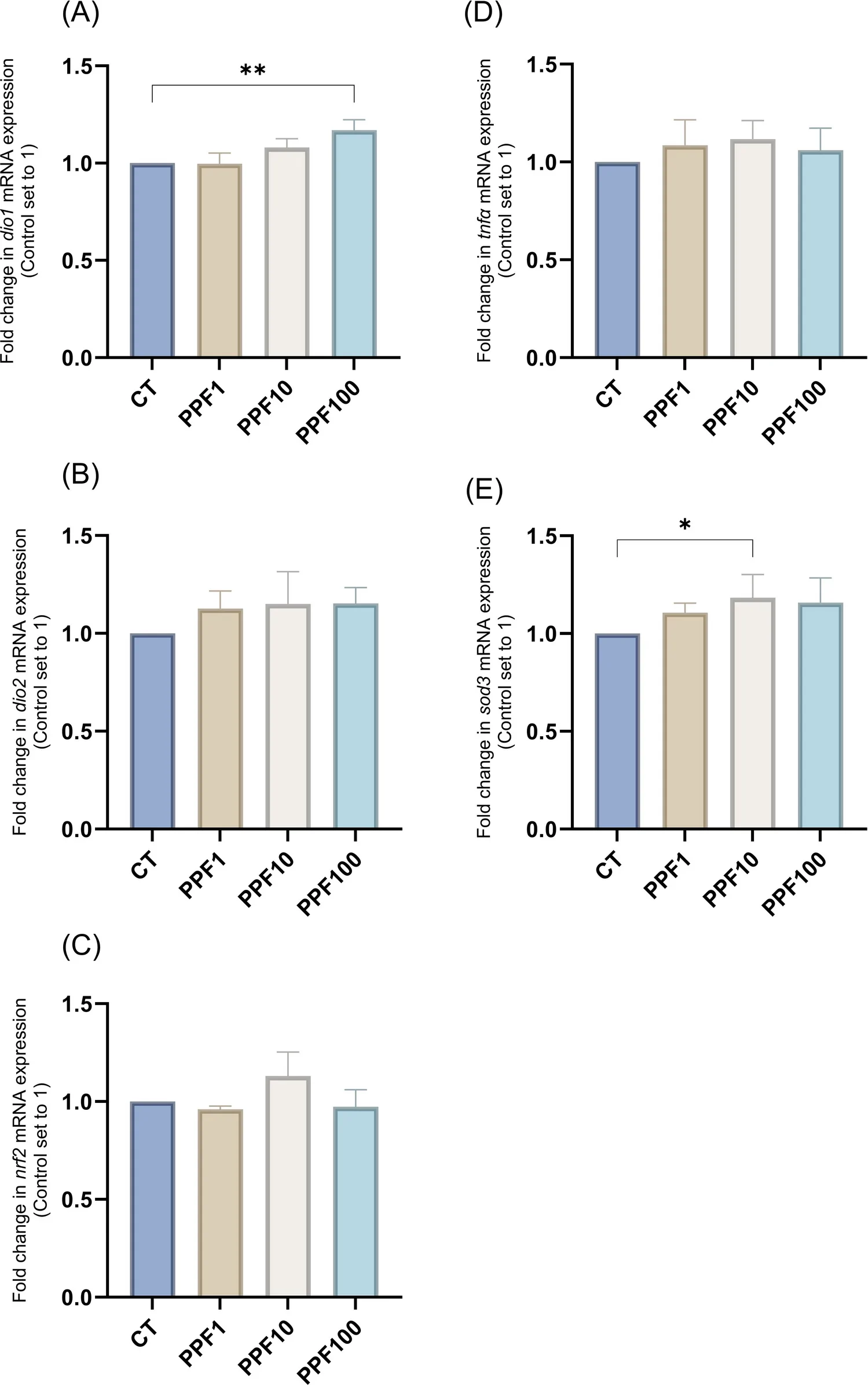
Relative mRNA expression of thyroid hormone metabolism- and stress-related genes in zebrafish liver after 14 days of exposure to PPF. The analyzed genes included (**a**) *dio1*, deiodinase type 1; (**b**) *dio2*, deiodinase type 2; (**c**) *nrf2*, nuclear factor erythroid 2-related factor 2; (**d**) *tnfα*, tumor necrosis factor alpha; and (**e**) *sod3*, superoxide dismutase 3. Data are presented as fold change relative to the control group, normalized to *actb1*. Results are expressed as the mean ± SEM of three independent biological replicates (n = 10 fish per replicate). Statistical significance was determined using one-way ANOVA followed by Dunnett's multiple comparisons test. Asterisks indicate significant differences compared with the control group (*p < 0.05; **p < 0.01; ***p < 0.001)

Given the pivotal role of the liver in thyroid hormone metabolism and oxidative homeostasis, we next examined the hepatic expression of deiodinases and stress-related genes in zebrafish exposed to PPF (Fig. 6). The expression of *dio1* was significantly upregulated in the PPF100 group (Fig. 6a), whereas *dio2* expression remained unchanged across treatments (Fig. 6b). Regarding oxidative and inflammatory markers, *nrf2* (Fig. 6c) and *tnfα* (Fig. 6d) did not show significant modulation; *sod3* expression was significantly increased in the intermediate group (Fig. 6e). These findings suggest that the highest PPF concentration may increase hepatic dio1 transcription without markedly affecting the other measured antioxidant or inflammatory markers.

### Renal tubular morphology was analyzed following PPF exposure

**Fig. 7:**
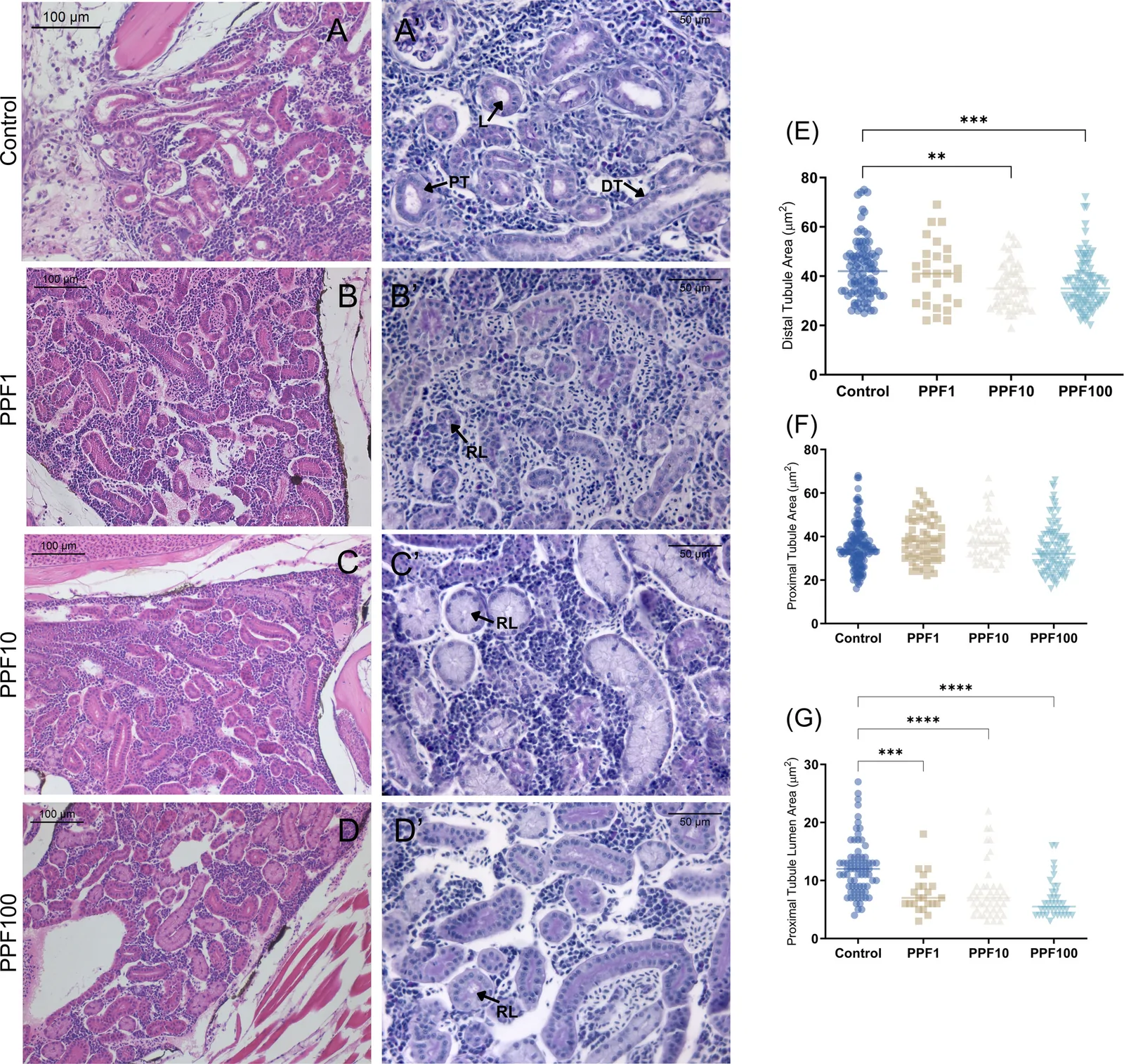
Histological and morphometric analysis of the kidney from adult female zebrafish after 14 days of PPF exposure. Representative photomicrographs of renal sections from (**a**, a') Control, (**b**, b') PPF1 (1 μg/L), (**c**, c') PPF10 (10 μg/L), and (**d**, d') PPF100 (100 μg/L) groups. Left panels show overall renal morphology stained with Hematoxylin & Eosin (scale bar = 100 μm); right panels show details of proximal and distal tubules stained with Periodic Acid–Schiff (PAS) (scale bar = 50 μm). Arrows in the high-magnification panels indicate PT, proximal tubule; DT, distal tubule; L, preserved proximal tubular lumen; and RL, reduced proximal tubular lumen. Morphometric measurements are shown for (**e**) distal tubule area, (**f**) proximal tubule area, and (**g**) proximal tubule lumen area in Control (CT) and PPF-exposed groups (1, 10, and 100 μg/L). Each point represents an individual measurement; bars indicate the mean. Statistical significance was determined using one-way ANOVA followed by Dunnett's multiple comparisons test. Asterisks denote significant differences compared with the control group (**p < 0.01; ***p < 0.001; ****p < 0.0001)

The kidney plays a crucial role in maintaining homeostasis and is a known target of endocrine-disrupting chemicals. Therefore, we next examined whether PPF exposure could affect renal structure in zebrafish. To this end, histological and morphometric analyses of proximal and distal tubules were performed after 14 days of exposure (Fig. 7).

Histological examination revealed alterations in tubular morphology compared with the control group. In control animals, proximal (PT) and distal tubules (DT) were well defined, exhibiting cuboidal epithelium and preserved lumens (L; Fig. 7a, a′). In PPF-exposed groups, reduced proximal tubular lumens (RL) were observed in the representative high-magnification sections (Fig. 7b′-d’).

These qualitative observations were supported by morphometric measurements. Distal tubular area (Fig. 7e) was significantly reduced in the PPF10 and PPF100 groups compared with controls, while no significant change was observed at PPF1. The proximal tubule area (Fig. 7f) showed no significant differences across groups. In contrast, the proximal tubule lumen area (Fig. 7g) was markedly decreased in all PPF-exposed groups (PPF1, PPF10 and PPF100) compared with controls. Together, these findings demonstrate that PPF exposure impairs renal tubular integrity in a concentration-dependent manner, particularly affecting the proximal tubule lumen.

## Discussion

PPF exposure induced concentration-specific changes in thyroid follicular morphology. Follicular epithelial cells mediate essential steps in thyroid hormone biosynthesis, whereas iodinated thyroglobulin is stored in the follicular colloid before hormone release (Carvalho and Dupuy 2017). Thus, the reductions in epithelial height at PPF1 and PPF10 and in follicle area at PPF1 indicate altered follicular architecture, but do not alone demonstrate impaired secretory function. At PPF10, epithelial thinning was accompanied by increased colloid area, suggesting an altered relationship between the follicular epithelium and the colloidal compartment; however, its functional significance remains uncertain because plasma T4 levels were unchanged.

These findings are biologically plausible considering previous evidence that PPF alters thyroid hormone-related gene expression in zebrafish and that its metabolite 4′-OH-PPF interferes with thyroid hormone signaling (Vancamp et al. 2021; Horie et al. 2023). At PPF100, irregular follicular profiles indicated qualitative architectural heterogeneity, although no significant morphometric differences were detected. Overall, the absence of a progressive effect with increasing PPF concentration is consistent with a non-monotonic concentration–response pattern, which has been described for endocrine-disrupting chemicals (Lagarde et al. 2015).

The marked increase in TSH levels at 100 µg/L, in the absence of parallel changes in T4, suggests a compensatory mechanism to sustain thyroid function, possibly reflecting primary thyroid dysfunction or altered peripheral sensitivity to thyroid hormones. Other studies have previously demonstrated alterations in thyroid hormone levels in different model organisms (Park et al. 2017; Vancamp et al. 2021).

Upregulation of *tpo* and *slc5a5* (nis), alongside downregulation of *tg*, suggests a compensatory molecular response to impaired hormone synthesis. Moreover, reduced *pax2a* expression indicates deficiencies in thyroid cell development and function in the group PPF100 (Carvalho and Dupuy 2017; Riesco-Eizaguirre et al. 2021). These observations align with previous reports demonstrating the sensitivity of the zebrafish HPT axis to various endocrine-disrupting chemicals.

In the brain, PPF exerted a complex, dose-dependent modulation of central HPT regulators. The downregulation of *thra* and *trhr* at 1 µg/L suggests an initial suppression of hypothalamic signaling and thyroid hormone responsiveness (Ortiga-Carvalho et al. 2016; Carvalho and Dupuy 2017). However, partial restoration of these genes at higher concentrations (10 and 100 µg/L), together with the upregulation of *trhr* at 10 µg/L and *tshb* at 100 µg/L, points to compensatory activation of the axis under sustained stress. Literature supports the complexity of HPT interactions with endocrine-disrupting chemicals, where concentration-dependent responses are frequently observed (Thambirajah et al. 2022; Mezzalira et al. 2026). For instance, Geng et al. (2023) reported that chronic cortisone exposure disrupts thyroid function and impairs retinal physiology in female zebrafish, underscoring the vulnerability of the HPT axis to xenobiotics. The ability of PPF to alter the expression of central regulators such as *trh* and *tshb* reinforces its role as a multi-level endocrine disruptor, with effects extending beyond the thyroid gland itself. Such central interference may have significant consequences for development and physiological regulation.

In the liver, a significant upregulation of *dio1* was observed at the highest PPF concentration (100 µg/L), while *dio2* remained unaffected. The increase in *dio1* transcription may reflect an adaptive attempt to fine-tune circulating thyroid hormone levels under disrupted conditions (Panicker et al. 2008; Marsili et al. 2011). Additionally, the marked upregulation of *sod3* at 10 µg/L indicates activation of antioxidant defenses and is consistent with a response to redox disturbance, although it does not by itself demonstrate oxidative damage (Wang et al. 2018). Li et al. (2022) and Robea et al. (2023) documented oxidative stress in fish exposed to PPF, reinforcing the significance of our observations. The absence of major changes in *nrf2* and *tnfα* suggests that although antioxidant defenses are activated, pro-inflammatory pathways may not be strongly triggered under the tested conditions, or that alternative stress-response mechanisms are involved. Even subtle hepatic dysfunction, however, may compromise detoxification processes and overall metabolic balance.

Renal histopathology further demonstrated that the kidney is a target organ for PPF toxicity. Significant reductions in the proximal tubule lumen area were observed across all PPF concentrations, while the distal tubule area was reduced at higher concentrations (10 and 100 μg/L), and no significant change was detected in the proximal tubule area. These findings suggest that, among the parameters examined, proximal tubule lumen area was the most consistently affected, with broader tubular alterations becoming evident at higher concentrations. Such structural alterations may potentially affect renal processes involved in osmoregulation and metabolite excretion. These findings are consistent with Gameiro et al. (2025), who reported epithelial degeneration in renal collecting ducts of adult male and female zebrafish after 96 h of exposure to 0.5–1.5 mg/L PPF. Compared with that qualitative acute assessment, the present study evaluated a longer, 14-day exposure at lower concentrations and provided quantitative morphometric evidence of alterations in proximal and distal tubular compartments. Renal impairment may also exacerbate thyroid dysfunction, given the kidney's role in thyroid hormone clearance and metabolism (Basu and Mohapatra 2012; Rhee 2016; Hafed et al. 2024). Collectively, the observed alterations in the thyroid, central HPT axis, liver, and kidney illustrate the systemic toxicity of PPF in zebrafish, with broad implications for organismal health and survival.

Disruption of the HPT axis, reflected by central and peripheral gene expression changes, has significant implications for zebrafish health (Jia et al. 2016; Parsons et al. 2020). The substantial genomic conservation between zebrafish and humans, together with the conservation of key components of thyroid hormone regulation, supports the mechanistic relevance of this model for investigating endocrine disruption (Opitz et al. 2011; Howe et al. 2013). However, the present findings should not be directly extrapolated to humans and require validation in mammalian and human-relevant experimental models. The modulation of hepatic antioxidant-response markers and the morphological alterations observed in the kidney highlight the systemic toxicity of PPF, which extends beyond its role as a juvenile hormone analog. Such dysfunctions may compromise the ability of organisms to cope with additional environmental stressors, reducing overall fitness and survival in contaminated habitats (Zhang et al. 2024; Cabral et al. 2024; Tominaga et al. 2025).

Previous multi-tissue studies have shown that PPF can affect several organs in fish. In adult zebrafish, acute exposure produced organ and sex-dependent histopathological effects across the liver, gills, intestine, kidneys, and gonads (Gameiro et al. 2025), whereas in *Labeo rohita*, PPF induced oxidative stress and DNA damage in blood, liver, kidneys, brain, and gills (Li et al. 2022). Against this background, the contribution of the present study is not the multi-tissue approach itself, but the integration of thyroid histology, circulating TSH and T4 measurements, central and peripheral HPT-axis gene expression, hepatic markers of thyroid hormone metabolism and antioxidant response, and quantitative renal morphometry in adult female zebrafish under the same exposure conditions. The discussion of non-monotonic responses and the proposal of alternative biomarkers represent novel contributions that may inform future research and refine environmental risk assessment strategies. The originality of this work also lies in the critical interpretation of experimental findings within a broader ecological and public health context.

## Conclusion

This study demonstrates that PPF exposure disrupts thyroid homeostasis and compromises systemic integrity in adult female zebrafish. A non-monotonic concentration–response was observed across thyroid endpoints, with the most pronounced molecular alterations occurring at 10 µg/L, the WHO reference application concentration for vector control in drinking-water containers. This pattern supports consideration of non-monotonic responses in future ecotoxicological risk assessment. The dissociation between unchanged T4 concentrations and marked histological and transcriptional disturbances also indicates that T4 alone may be insufficient to detect early PPF-induced thyroid dysfunction. Future studies should integrate broader endocrine and metabolic markers, including T3, rT3, deiodinase activity, and genes related to hormone transport and metabolism. Collectively, these findings support integrative strategies that evaluate endocrine and renal endpoints while accounting for the limitations of direct cross-species extrapolation.

## Supplementary Information

Below is the link to the electronic supplementary material.

Supplementary file1 Supplementary material Primer sequences used for qPCR in Danio rerio (zebrafish). Gene abbreviations: actb1 (β-actin, housekeeping gene), trhr (thyrotropin-releasing hormone receptor), trh (thyrotropin-releasing hormone), tshb (thyroid-stimulating hormone beta subunit), tshr (thyroid-stimulating hormone receptor), thra (thyroid hormone receptor alpha), slc5a5 (nis) (sodium/iodide symporter), tg (thyroglobulin), tpo (thyroid peroxidase), pax2a/pax2.1 (paired box gene 2a, thyroid transcription factor), nkx2.1 (NK2 homeobox 1), nrf2 (nuclear factor erythroid 2–related factor 2), tnfα (tumor necrosis factor alpha), sod3 (superoxide dismutase 3), dio1 (deiodinase type 1), dio2 (deiodinase type 2). (PDF 165 KB)

## Data Availability

Data will be made available on reasonable request.

## References

[CR1] Abdu U, Barki A, Karplus I et al (2001) Physiological effects of methyl farnesoate and pyriproxyfen on wintering female crayfish *Cherax quadricarinatus*. Aquaculture 202:163–175. 10.1016/S0044-8486(01)00596-8

[CR2] Adhish M, Manjubala I (2023) Effectiveness of zebrafish models in understanding human diseases: a review of models. Heliyon 9:e14557. 10.1016/j.heliyon.2023.e14557 PMC10025926 36950605

[CR3] Ahn C, Jeung E-B (2023) Endocrine-disrupting chemicals and disease endpoints. Int J Mol Sci 24:5342. 10.3390/ijms24065342 PMC10049097 36982431

[CR4] Alomar AA, Alto BW (2022) Evaluation of pyriproxyfen effects on *Aedes aegypti* and predatory mosquito *Toxorhynchites rutilus* (Diptera: Culicidae). J Med Entomol 59:585–590. 10.1093/jme/tjab193 34865101

[CR5] American Veterinary Medical Association (2020) AVMA Guidelines for the Euthanasia of Animals: 2020 Edition. American Veterinary Medical Association, Schaumburg, IL. https://www.avma.org/resources-tools/avma-policies/avma-guidelines-euthanasia-animals

[CR6] Azeredo DBC, de Sousa AD, Veríssimo ACF et al (2025) Endocrine-disrupting chemical, methylparaben, in environmentally relevant exposure promotes hazardous effects on the hypothalamus-pituitary-thyroid axis. Mol Cell Endocrinol 598:112444. 10.1016/j.mce.2024.112444 39725349

[CR7] Basu G, Mohapatra A (2012) Interactions between thyroid disorders and kidney disease. Indian J Endocrinol Metab 16:204–213. 10.4103/2230-8210.93737 PMC3313737 22470856

[CR8] Baumann L, Ros A, Rehberger K et al (2016) Thyroid disruption in zebrafish (*Danio rerio*) larvae: different molecular response patterns lead to impaired eye development and visual functions. Aquat Toxicol 172:44–55. 10.1016/j.aquatox.2015.12.015 26765085

[CR9] Bianco AC, Anderson G, Forrest D et al (2014) American Thyroid Association guide to investigating thyroid hormone economy and action in rodent and cell models: report of the American Thyroid Association Task Force on Approaches and Strategies to investigate thyroid hormone economy and action. Thyroid 24:88–168. 10.1089/thy.2013.0109 PMC3887458 24001133

[CR10] Cabral AP, Maia FPDS, Magliano DC, Graceli JB, Soares P, Morris EAR, Miranda-Alves L (2024) Pyriproxyfen: villain or good guy? A brief review. Arch Endocrinol Metab 68:e240154. 10.20945/2359-4292-2024-0154 PMC11771759 39876972

[CR11] Carvalho DP, Dupuy C (2017) Thyroid hormone biosynthesis and release. Mol Cell Endocrinol 458:6–15. 10.1016/j.mce.2017.01.038 28153798

[CR12] Cazarin J, Dupuy C, de Carvalho DP (2022) Redox homeostasis in thyroid cancer: implications for Na+/I− symporter (NIS) regulation. Int J Mol Sci 23:6129. 10.3390/ijms23116129 PMC9181215 35682803

[CR13] Chiamolera MI, Sidhaye AR, Matsumoto S et al (2012) Fundamentally distinct roles of thyroid hormone receptor isoforms in a thyrotroph cell line are due to differential DNA binding. Mol Endocrinol 26:926–939. 10.1210/me.2011-1290 PMC3355539 22570333

[CR14] Christou M, Kavaliauskis A, Ropstad E, Fraser TWK (2020) DMSO affects larval zebrafish (*Danio rerio*) behavior, with additive and interaction effects when combined with positive controls. Sci Total Environ 709:134490. 10.1016/j.scitotenv.2019.134490 31905542

[CR15] Devillers J (2020a) Fate of pyriproxyfen in soils and plants. Toxics 8:20. 10.3390/toxics8010020 PMC7151735 32183189

[CR16] Devillers J (2020b) Fate and ecotoxicological effects of pyriproxyfen in aquatic ecosystems. Environ Sci Pollut Res 27:16052–16068. 10.1007/s11356-020-08345-8 32180143

[CR17] Egalini F, Marinelli L, Rossi M et al (2022) Endocrine-disrupting chemicals: effects on pituitary, thyroid and adrenal glands. Endocr Res 78:395–405. 10.1007/s12020-022-03076-x PMC9637063 35604630

[CR18] Ely KA, Bischoff LA, Weiss VL (2018) Wnt signaling in thyroid homeostasis and carcinogenesis. Genes 9:204. 10.3390/genes9040204 PMC5924546 29642644

[CR19] European Parliament and Council of the European Union (2010) Directive 2010/63/EU of the European Parliament and of the Council of 22 September 2010 on the protection of animals used for scientific purposes. Official Journal of the European Union L276:33–79. https://eur-lex.europa.eu/legal-content/EN/TXT/?uri=CELEX:32010L0063

[CR20] Gameiro BG, Brito RS, Valle ADA et al (2025) Acute toxicity and histopathological effects of pyriproxyfen in adult male and female zebrafish (*Danio rerio*). Environ Sci Adv 4:1117–1128. 10.1039/D4VA00380B

[CR21] Geng Y, Zou H, Guo Y, Huang M, Wu Y, Hou L (2023) Chronic exposure to cortisone induces thyroid endocrine disruption and retinal dysfunction in adult female zebrafish (*Danio rerio*). Sci Total Environ 905:167022. 10.1016/j.scitotenv.2023.167022 37709101

[CR22] Gereben B, Zavacki AM, Ribich S et al (2008) Cellular and molecular basis of deiodinase-regulated thyroid hormone signaling. Endocr Rev 29:898–938. 10.1210/er.2008-0019 PMC2647704 18815314

[CR23] Ginjupalli GK, Baldwin WSB (2013) Time- and age-dependent effects of the juvenile hormone analog pesticide pyriproxyfen on *Daphnia magna* reproduction. Chemosphere 92:1260–1266. 10.1016/j.chemosphere.2013.04.061 PMC3716864 23714148

[CR24] Grisales N, Lees RS, Maas J et al (2021) Pyriproxyfen-treated bed nets reduce reproductive fitness and longevity of pyrethroid-resistant *Anopheles gambiae* under laboratory and field conditions. Malar J 20:273. 10.1186/s12936-021-03794-z PMC8218427 34158066

[CR25] Hafed AB, Abdulkareem RK, Almalki AM et al (2024) Assessment of thyroid function in chronic kidney disease patients at King Abdulaziz Medical City. Cureus 16:e73180. 10.7759/cureus.73180 PMC11624967 39650928

[CR26] He J, Xu J, Zheng M et al (2024) Thyroid dysfunction caused by exposure to environmental endocrine disruptors and the underlying mechanism: a review. Chem Biol Interact 391:110909. 10.1016/j.cbi.2024.110909 38340975

[CR27] Horie Y, Mitsunaga K, Yap CK (2023) Pyriproxyfen influences growth and thyroid hormone-related and gh/igf-1 gene expression during early life stages of zebrafish (*Danio rerio*). Comp Biochem Physiol C Toxicol Pharmacol 269:109632. 10.1016/j.cbpc.2023.109632 37075951

[CR28] Howe K, Clark MD, Torroja CF et al (2013) The zebrafish reference genome sequence and its relationship to the human genome. Nature 496:498–503. 10.1038/nature12111 PMC3703927 23594743

[CR29] Hoyberghs J, Bars C, Ayuso M et al (2021) DMSO concentrations up to 1% are safe to be used in the zebrafish embryo developmental toxicity assay. Front Toxicol 3:804033. 10.3389/ftox.2021.804033 PMC8915880 35295145

[CR30] Hustedt JC, Boyce R, Bradley J et al (2020) Use of pyriproxyfen in control of *Aedes* mosquitoes: a systematic review. PLoS Negl Trop Dis 14:e0008205. 10.1371/journal.pntd.0008205 PMC7314096 32530915

[CR31] Hustedt JC, Doum D, Keo V et al (2021) Field efficacy of larvivorous fish and pyriproxyfen combined with community engagement on dengue vectors in Cambodia: a randomized controlled trial. Am J Trop Med Hyg 105:1265–1276. 10.4269/ajtmh.20-1088 PMC8592206 34491225

[CR32] Ihn Y, Cho Y, Lee Y et al (2024) Thyroid and sex hormone-disrupting effects of DEHTP at different life stages of zebrafish (*Danio rerio*). Chemosphere 358:142105. 10.1016/j.chemosphere.2024.142105 38657690

[CR33] Ikegami K, Yoshimura T (2017) The hypothalamic–pituitary–thyroid axis and biological rhythms: discovery of TSH’s unexpected role using animal models. Best Pract Res Clin Endocrinol Metab 31:475–485. 10.1016/j.beem.2017.09.002 29223282

[CR34] Jia P-P, Ma Y-B, Lu C-J et al (2016) Disturbance of the hypothalamus–pituitary–thyroid axis in zebrafish larvae after DEHP exposure. PLoS ONE 11:e0155762. 10.1371/journal.pone.0155762 PMC4880181 27223697

[CR35] Jiao F, Qiao K, Jiang Y et al (2019) Integrated thyroid endocrine disrupting effect on zebrafish (*Danio rario*) larvae via simultaneously repressing type II iodothyronine deiodinase and activating thyroid receptor-mediated signaling following waterborne exposure to trace azocyclotin. Environ Pollut 255:113328. 10.1016/j.envpol.2019.113328 31671316

[CR36] Jugan M-L, Levi Y, Blondeau J (2010) Endocrine disruptors and thyroid hormone physiology. Biochem Pharmacol 79:939–947. 10.1016/j.bcp.2009.11.006 19913515

[CR37] Kamarudin NA, Zulkifli SZ, Azmai MNA et al (2020) Herbicide diuron as an endocrine-disrupting chemical assessed by histopathological analysis in gonads of Javanese medaka (Oryzias javanicus). Animals 10:525. 10.3390/ani10030525 PMC7142969 32245133

[CR38] Köhrle J (2000) The deiodinase family: selenoenzymes regulating thyroid hormone availability and action. Cell Mol Life Sci 57:1853–1863. 10.1007/PL00000667 PMC11147027 11215512

[CR39] Köhrle J, Frädrich C (2022) Deiodinases control local cellular and systemic thyroid hormone availability. Free Radic Biol Med 193:59–79. 10.1016/j.freeradbiomed.2022.09.024 36206932

[CR40] Kumari P, Duhan A, Rani N, Tomar D (2021) Ultimate fate and toxicological consequences of the insecticide pyriproxyfen and its metabolites in soil ecosystems. Environmental Advances 4:100040. 10.1016/j.envadv.2021.100040

[CR41] Lagarde F, Beausoleil C, Belcher SM et al (2015) Non-monotonic dose-response relationships and endocrine disruptors: a qualitative method of assessment. Environ Health 14:13. 10.1186/1476-069X-14-13 PMC4429934 25971433

[CR42] Lee S, Eghan K, Lee J et al (2020) Zebrafish embryonic exposure to BPAP and its relatively weak thyroid hormone-disrupting effects. Toxics 8:103. 10.3390/toxics8040103 PMC7712898 33202880

[CR43] Li X, Naseem S, Hussain R et al (2022) Evaluation of DNA damage, biomarkers of oxidative stress, and status of antioxidant enzymes in freshwater fish (*Labeo rohita*) exposed to pyriproxyfen. Oxid Med Cell Longev. 10.1155/2022/5859266 PMC9205694 35720182

[CR44] Livak KJ, Schmittgen TD (2001) Analysis of relative gene expression data using real-time quantitative PCR and the 2^−ΔΔCT^ method. Methods 25:402–408. 10.1006/meth.2001.1262 11846609

[CR45] Luongo C, Dentice M, Salvatore D (2019) Deiodinases and their intricate role in thyroid hormone homeostasis. Nat Rev Endocrinol 15:479–488. 10.1038/s41574-019-0218-2 31160732

[CR46] Maharajan K, Muthulakshmi S, Karthik C et al (2020) Pyriproxyfen induced impairment of reproductive endocrine homeostasis and gonadal histopathology in zebrafish (*Danio rerio*) by altered expression of hypothalamus-pituitary-gonadal (HPG) axis genes. Sci Total Environ 735:139496. 10.1016/j.scitotenv.2020.139496 32480152

[CR47] Marsili A, Zavacki AM, Harney JW, Larsen PR (2011) Physiological role and regulation of iodothyronine deiodinases: a 2011 update. J Endocrinol Invest 34:395–407. 10.1007/BF03347465 PMC3687787 21427525

[CR48] Megson D, Reiner EJ, Jobst KJ, Dorman FL, Robson M, Focant J-F (2016) A review of the determination of persistent organic pollutants for environmental forensics investigations. Anal Chim Acta 941:10–25. 10.1016/j.aca.2016.08.027 27692373

[CR49] Mezzalira N, Rodrigues VG, Serrano-Nascimento C (2026) Zebrafish: a key model for unraveling endocrine-disrupting effects on thyroid development and function. Front Pharmacol 17:1746017. 10.3389/fphar.2026.1746017 PMC12957125 41788796

[CR50] Monteiro FJC, Mourão FRP, Ribeiro ESD, Rêgo MOS, Frances PAC, Souto RNP, Façanha MS, Tahmasebi R, Costa ACD (2020) Prevalence of dengue, Zika and chikungunya viruses in *Aedes aegypti* in a medium-sized city in the Amazon, Brazil. Rev Inst Med Trop São Paulo 62:e10. 10.1590/S1678-9946202062010 PMC7014551 32049261

[CR51] Moura JAS, Souza-Santos LP (2020) Environmental risk assessment of pyriproxyfen in non-target aquatic organisms. Aquat Toxicol 222:105448. 10.1016/j.aquatox.2020.105448 32197184

[CR52] National Council for the Control of Animal Experimentation (CONCEA) (2017) Brazilian guideline for the care and use of animals for scientific and educational purposes [in Portuguese]. Ministry of Science, Technology and Innovations, Brasília, DF, Brazil. http://www.mctic.gov.br/mctic/opencms/institucional/concea/arquivos/legislacao/resolucoes_normativas/Resolucao-Normativa-CONCEA-n-34-de-27.07.2017-D.O.U.-de-31.07.2017-Secao-I-Pag.-218-Anexo.pdf

[CR53] Opitz R, Maquet E, Zoenen M, Dadhich R, Costagliola S (2011) TSH receptor function is required for normal thyroid differentiation in zebrafish. Mol Endocrinol 25:1579–1599. 10.1210/me.2011-0046 PMC5417235 21737742

[CR54] Ortiga-Carvalho TM, Chiamolera MI, Pazos-Moura CC et al (2016) Hypothalamus–pituitary–thyroid axis. Compr Physiol 6:1387–1428. 10.1002/cphy.c150027 27347897

[CR55] Paiva-Melo FD, de Sousa AD, Teixeira MP et al (2025) The hypothalamus–pituitary–thyroid axis is disrupted by exposure to a mixture of tributyltin and bisphenol S. Environ Pollut 368:125754. 10.1016/j.envpol.2025.125754 39884547

[CR56] Panicker V, Cluett C, Shields B et al (2008) A common variation in the deiodinase 1 gene (DIO1) is associated with relative levels of free thyroxine and triiodothyronine. J Clin Endocrinol Metab 93:3075–3081. 10.1210/jc.2008-0397 PMC2515080 18492748

[CR57] Park GC, Kim JM, Park H-Y et al (2017) TSH-independent release of thyroid hormones through cold exposure in aging rats. Oncotarget 8:89431–89438. 10.18632/oncotarget.19851 PMC5685681 29163760

[CR58] Parsons AE, Lange A, Hutchinson TH et al (2020) Expression dynamics of genes in the hypothalamic-pituitary-thyroid (HPT) cascade and their responses to 3,3′,5-triiodo-l-thyronine (T3) highlights potential vulnerability to thyroid-disrupting chemicals in zebrafish *(Danio rerio*) embryo-larvae. Aquat Toxicol 225:105547. 10.1016/j.aquatox.2020.105547 32623180

[CR59] Paul B, Sterner ZR, Buchholz DR et al (2022) Thyroid and corticosteroid signaling in amphibian metamorphosis. Cells 11:1595. 10.3390/cells11101595 PMC9139329 35626631

[CR60] Pereira LC, Souza AO, Bernardes MFF et al (2015) A perspective on the potential risks of emerging contaminants to human and environmental health. Environ Sci Pollut Res 22:13800–13823. 10.1007/s11356-015-4896-6 26201652

[CR61] Porto VA, Rocha Júnior ER, Ursulino JS et al (2023) NMR-based metabolomics applied to ecotoxicology with zebrafish (*Danio rerio*) as a prominent model for metabolic profiling and biomarker discovery: overviewing the most recent approaches. Sci Total Environ 868:161737. 10.1016/j.scitotenv.2023.161737 36693575

[CR62] Raza A, Tasleem S, Ullah N, Dilshad M, Maitlo AA (2024) Quantifying heavy metal accumulation in soil and vegetation surrounding industrial areas: a comprehensive environmental toxicology study. J Health Rehabil Res. 10.61919/jhrr.v4i2.864

[CR63] Rhee CM (2016) The interaction between thyroid and kidney disease: an overview of the evidence. Curr Opin Endocrinol Diabetes Obes 23:407–415. 10.1097/MED.0000000000000275 PMC5094898 27428519

[CR64] Riesco-Eizaguirre G, Santisteban P, De la Vieja A (2021) Regulation of NIS expression and activity in thyroid and extrathyroidal tissues. Endocr Relat Cancer 28:T141–T165. 10.1530/ERC-21-0217 34387194

[CR65] Río Barrera T, Zambrano Ledesma KN, Aguilar Hernández M et al (2025) Endocrine disruptors and their impact on quality of life: a literature review. Cureus 17:e83890. 10.7759/cureus.83890 PMC12066167 40351481

[CR66] Robea MA, Petrovici A, Ureche D, Nicoara M, Ciobica AS (2023) Histopathological and behavioral impairments in zebrafish chronically exposed to fipronil and pyriproxyfen. Life 13:1874. 10.3390/life13091874 PMC10533071 37763278

[CR67] Russo SC, Salas-Lucia F, Bianco AC (2021) Deiodinases and the metabolic code for thyroid hormone action. Endocrinology 162:bqab059. 10.1210/endocr/bqab059 PMC8237994 33720335

[CR68] Sabatino L, Vassalle C, Seppia CD, Iervasi G (2021) Deiodinases and the three types of thyroid hormone deiodination reactions. Endocrinol Metab 36:952–964. 10.3803/EnM.2021.1198 PMC8566136 34674502

[CR69] Schmutzler C, Gotthardt I, Hofmann PJ et al (2007) Endocrine disruptors and the thyroid gland: combined in vitro and in vivo analysis of potential biomarkers. Environ Health Perspect 115(Suppl 1):77–83. 10.1289/ehp.9369 PMC2174406 18174954

[CR70] Segner H (2009) Zebrafish (*Danio rerio*) as a model organism for investigating endocrine disruption. Comp Biochem Physiol C 149:187–195. 10.1016/j.cbpc.2008.10.099 18955160

[CR71] Teame T, Zhang Z, Ran C et al (2019) The use of zebrafish (*Danio rerio*) as biomedical models. Anim Front 9:68–77. 10.1093/af/vfz020 PMC6951987 32002264

[CR72] Thambirajah AA, Wade MG, Verreault J et al (2022) Disruption by stealth - interference of endocrine disrupting chemicals on hormonal crosstalk with thyroid axis function in humans and other animals. Environ Res 203:111906. 10.1016/j.envres.2021.111906 34418447

[CR73] Tominaga FK, Brito RS, Oliveira Nascimento J et al (2025) Pyriproxyfen toxicity to fish and crustaceans: a literature review. Environ Res 274:121295. 10.1016/j.envres.2025.121295 40049357

[CR74] Truong L, Gonnerman G, Simonich MT, Tanguay RL (2016) Assessment of the developmental and neurotoxicity of the mosquito control larvicide, pyriproxyfen, using embryonic zebrafish. Environ Pollut 218:1089–1093. 10.1016/j.envpol.2016.08.061 PMC5048575 27593350

[CR75] Tudi M, Ruan HD, Wang L et al (2021) Agriculture development, pesticide application and its impact on the environment. Int J Environ Res Public Health 18:1112. 10.3390/ijerph18031112 PMC7908628 33513796

[CR76] Ur Rahman A, Khan I, Usman A, Khan H (2024) Evaluation of insect growth regulators (igrs) as biological pesticides for Aedes aegypti mosquitoes. J Vector Borne Dis 61:129–135. 10.4103/0972-9062.392257 38648415

[CR77] Vancamp P, Spirhanzlova P, Sébillot A et al (2021) The pyriproxyfen metabolite 4′-OH-PPF disrupts thyroid hormone signaling in neural stem cells, modifying neurodevelopmental genes affected by Zika virus infection. Environ Pollut 285:117654. 10.1016/j.envpol.2021.117654 34289950

[CR78] Wade MG, Parent S, Finnson KW et al (2002) Thyroid toxicity due to subchronic exposure to a complex mixture of organochlorines, lead and cadmium. Toxicol Sci 67:207–218. 10.1093/toxsci/67.2.207 12011480

[CR79] Wang Y, Branicky R, Noë A, Hekimi S (2018) Superoxide dismutases: dual roles in controlling ROS damage and regulating ROS signaling. J Cell Biol 217:1915–1928. 10.1083/jcb.201708007 PMC5987716 29669742

[CR80] Wei Y, Cui J, Zhai W et al (2021) Toxicity and fate of chiral insecticide pyriproxyfen and its metabolites in zebrafish (*Danio rerio*). Environ Pollut 280:116894. 10.1016/j.envpol.2021.116894 33774544

[CR81] World Health Organization (2008) Pyriproxyfen in drinking-water: use for vector control in drinking-water sources and containers. Background document for development of WHO Guidelines for Drinking-water Quality. WHO, Geneva. WHO/HSE/AMR/08.03/9

[CR82] Wu C, Liu X, He M et al (2021) Determination of pyriproxyfen and its metabolites in bee products using modified QuEChERS and UPLC–MS/MS. Ecotoxicol Environ Saf 220:112388. 10.1016/j.ecoenv.2021.112388 34091183

[CR83] Xu Z, Zheng Q, Li N et al (2024) Rational design of a dual-mode fluorescent probe for portable detection of pyriproxyfen in the environment and food. J Hazard Mater 477:135364. 10.1016/j.jhazmat.2024.135364 39111178

[CR84] Yadav K, Dhiman S, Acharya BN, Ghorpade RR, Sukumaran D (2019) Pyriproxyfen-treated surfaces induce reproductive disruption in dengue vector *Aedes aegypti*. PLoS Negl Trop Dis 13:e0007842. 10.1371/journal.pntd.0007842 PMC6886876 31738760

[CR85] Yilmaz B, Terekeci H, Sandal S, Kelestimur F (2020) Endocrine-disrupting chemicals: exposure, effects on human health, mechanisms of action, testing models and prevention strategies. Rev Endocr Metab Disord 21:127–147. 10.1007/s11154-019-09521-z 31792807

[CR86] Zhang F-Y, Fang Y, Zhang C-X, Zhang H-Y, Dong M, Zhang K-W, Wu C, Song H-D, Chen G (2024) The effects of disturbance on the hypothalamus–pituitary–thyroid axis in zebrafish larvae after exposure to polyvinyl alcohol. Ecotoxicol Environ Saf 288:117396. 10.1016/j.ecoenv.2024.117396 39603223

