# Supplementary material for: Endocrine and systemic toxicity of pyriproxyfen in adult female zebrafish: disruption of the HPT axis and peripheral organ impairments
Source: Environ Sci Pollut Res Int. 2026 Oct 19;33(37):17497–512. doi: 10.1007/s11356-026-38319-1 (PMC13719738; doi:10.1007/s11356-026-38319-1)
Supplement: Supplementary file 1 [file 11356_2026_38319_MOESM1_ESM.pdf]

Table S1. Primer sequences used for qPCR in *Danio rerio* (zebrafish)

**Gene (symbol)**	**Forward (5'–3')**	**Reverse (5'–3')**
actb1 (housekeeping)	CGAGCAGGAGATGGGAACC	CAACGGAAACGCTCATTGC
trhr	CTGGTGGTGTCAACTCCTT	GCTTTCCACCGTTGATGTTT
trh	CACACAGATGGAGGAGCAGA	AGCAGCATCAGGTAGCGTTT
tshb	GCAGATCCTCACTTCACCTACC	GCACAGGTTTGGAGCATCTCA
tshr	GCTCCTTGATGTGTCCGAAT	CGGGCAGTCAGGTTACAAAT
thra	CTATGAACAGCACATCCGACAAG	CACACCACACACGGCTCATC
slc5a5 (nis)	GGTGGCATGAAGGCTGTAAT	GATACGGCATCCATTGTTGG
tg	CCAGCCGAAAGGATAGAGTTG	ATGCTGCCGTGGAATAGGA
tpo	AAGGGATGTTCCTGTTTCTGT	GAAGGGTTCGGGATTCATTAC
pax2a (pax2.1)	AACCCCGCGTTATTAAGTTC	GCGTATCCATCTTCAATCCC
nkx2.1	TGTACAAAACCACCATCACC	CCTACCTGCTGACTTTGATG
nrf2	GTGAACAACCCTCCGATTAC	CTTGCATCTAGGGACAAACC
tnfα	TATGAAGCTTGAGAGTCGGGCGTT	AAATGGATGGCAGCCTTGGAAGTG
sod3	GCCTCGTGTGTATGGTCACA	TGGATGGTTGACGTTGAGGG
dio1	GTTCAAACAGCTTGTCAAGGACT	AGCAAGCCTCTCCTCCAAGTT
dio2	GCATAGGCAGTCGCTCATTT	TGTGGTCTCTCATCCAACCA
